# Moving Forward: Recent Developments for the Ferret Biomedical Research Model

**DOI:** 10.1128/mBio.01113-18

**Published:** 2018-07-17

**Authors:** Randy A. Albrecht, Wen-Chun Liu, Andrea J. Sant, S. Mark Tompkins, Andrew Pekosz, Victoria Meliopoulos, Sean Cherry, Paul G. Thomas, Stacey Schultz-Cherry

**Affiliations:** a Department of Microbiology, Icahn School of Medicine at Mount Sinai, New York, New York, USA; b David H. Smith Center for Vaccine Biology and Immunology, Department of Microbiology and Immunology, University of Rochester Medical Center, Rochester, New York, USA; c Center for Vaccines and Immunology, University of Georgia, Athens, Georgia, USA; d W. Harry Feinstone Department of Molecular Microbiology and Immunology, Bloomberg School of Public Health, Johns Hopkins University, Baltimore, Maryland, USA; e Department of Immunology, St. Jude Children’s Research Hospital, Memphis, Tennessee, USA; f Department of Infectious Diseases, St. Jude Children’s Research Hospital, Memphis, Tennessee, USA; University of Colorado School of Medicine

**Keywords:** advances, animal model, ferret

## Abstract

Since the initial report in 1911, the domestic ferret has become an invaluable biomedical research model. While widely recognized for its utility in influenza virus research, ferrets are used for a variety of infectious and noninfectious disease models due to the anatomical, metabolic, and physiological features they share with humans and their susceptibility to many human pathogens. However, there are limitations to the model that must be overcome for maximal utility for the scientific community. Here, we describe important recent advances that will accelerate biomedical research with this animal model.

## Abstract (precis)

Since the initial report in 1911, the domestic ferret has become an invaluable biomedical research model. While widely recognized for its utility in influenza virus research, ferrets are used for a variety of infectious and noninfectious disease models due to the anatomical, metabolic, and physiological features they share with humans and their susceptibility to many human pathogens.

## INTRODUCTION

In 1911, the first study using the domestic ferret, Mustela putorius *furo*, for biomedical research was published (1). Since then, the ferret has been an invaluable model for cardiac research (2), spinal cord injury (3), epilepsy (4), and several lung conditions, including smoke-induced chronic obstructive pulmonary disease (COPD) (5), cystic fibrosis (6), and tobacco-induced lung cancer (7). The recent development of a database of the anatomical connections and structural features of the ferret brain will likely also improve the relevance of this model for neurological research (8). Yet, the ferret model is most widely recognized for its utility in infectious disease research, especially respiratory infections (Table 1). A variety of human pathogens are known to naturally infect ferrets and often reproduce human disease better than mouse models. In this article, we discuss the recent advances and ongoing initiatives to increase the utility of the ferret model for biomedical research.

**TABLE 1:** Human microbes used in the ferret model

Pathogen group and species	Reference(s)
Viruses	
Influenza virus	26
Respiratory syncytial virus	27, 28
*Metapneumovirus*	29
Measles virus	30
Mumps virus	31, 32
Parainfluenza viruses	33, 34
Severe acute respiratory syndrome coronavirus	35
Nipah virus	36
Ebola virus	37
Rift Valley fever virus	38
Bacteria	
*Streptococcus* spp.	39
Staphylococcus aureus	40
Helicobacter mustelae	41
*Mycobacterium* spp.	42
Fungi	
Pneumocystis jirovecii	43

## MODELS, GENOMES, AND OMICS

The first transgenic ferret was produced by somatic cell nuclear transfer (SCNT) to oocyte recipient cells in 2006 (9). This technique was then combined with adeno-associated virus-mediated gene targeting of the cystic fibrosis transmembrane conductance regulator (CFTR) gene to generate a transgenic ferret model of cystic fibrosis and create the first reported ferret genomic bacterial artificial chromosome library (10). More recently, CRISPR/Cas9-mediated genome editing techniques were applied to ferrets to develop a model organism to study X-linked, double cortin-related lissencephaly spectrum (11). In addition to genetically modified ferrets, research groups have described the development of immunocompromised (12), pregnant (13), aged (14), and diet-induced obese (DIO [unpublished data]) models to understand disease in high-risk populations. It is likely that new models and transgenic animals will be developed in the near future.

The sequencing of the ferret genome (15) was instrumental in advancing functional genomic analysis. Numerous groups developed reagents to monitor gene-specific mRNA expression levels via TaqMan-based or Sybr green-based real-time reverse transcription-PCR assays for a plethora of targets. Many of these primers are available free of charge through the National Institute of Allergy and Infectious Diseases (NIAID) established BEI Resources (https://www.beiresources.org/Home.aspx). Bruder et al. described the development of an expression microarray platform that included the identification of 41 genes with consistent baseline transcription profiles across tissues that could be used as housekeeping genes (16). Our group developed and is validating a FLUIDIGM panel with 144 distinct immune response and lung injury and repair genes. Beyond transcription, Tisoncik-Go et al. described an integrated omics analysis that profiles lipids, metabolites, and proteins in the respiratory compartments of influenza virus-infected ferrets (17). Combined, these tools provide powerful resources to the research community.

## THE NEXT FRONTIER: THE IMMUNE RESPONSE

Despite its relevance for biomedical research, there are limitations of the ferret model for immunologic studies due to the dearth of reagents. Screening of commercially available antibodies for cross-reactivity with markers on innate and adaptive cell subsets and cytokines in ferrets has yielded limited success (Table 2). To resolve this, a group of researchers from around the world are working together to develop validated reagents and assays to improve our understanding of the innate and adaptive immune responses in the ferret.

**TABLE 2:** Commercial kits and immunologic reagents tested in the ferret model

Product type and name^a^	Specificity	Clone	Isotype	Host	Vendor	Application	Reference(s)
Commercial kits							
LIVE/ DEAD Fixable Aqua dead cell stain					Thermo/Fisher	Flow cyt	18
IFN-γ ELISpot basic (HRP) kit					MabTech	ELISpot	18
Primary antibodies							
CD44	Mouse	IM7	IgG2b, κ	Rat	BD Pharmingen	Flow cyt	19
IL-4	Bovine	CC303	IgG2a	Mouse	Bio-Rad	Flow cyt	19
IFN-γ	Bovine	CC302	IgG1	Mouse	Bio-Rad	Flow cyt	19
IFN-γ	Mouse	XMG1.2	IgG1, κ	Rat	BD Pharmingen	Flow cyt	19
TNF	Mouse	MP6-XT22	IgG1	Rat	BD Pharmingen	Flow cyt	19
Thy1.1	Rat	OX-7	IgG1, κ	Mouse	BD Pharmingen	Flow cyt	19
CD11b	Mouse/human	M1/70	IgG2b, κ	Rat	BD Pharmingen or BioLegend	Flow cyt	18, 19
CD8a	Human	OK-T8	IgG2a	Mouse	eBioscience/Tonbo	Flow cyt	18, 19
CD4	Ferret	02	IgG1	Mouse	Sino Biological	Flow cyt	18, 19
MHC-II	Human	L243	IgG2a, κ	Mouse	BioLegend	Flow cyt	18
IgA, IgM, IgG	Ferret		Poly	Goat	LSBio	Flow cyt	18
CD59	Mouse	AL-21	IgM, κ	Rat	BD Pharmingen	Flow cyt	18
CD79a	Human	HM47	IgG1, κ	Mouse	eBioscience	Flow cyt	18
CD20	Ferret	71	IgG	Rabbit	Sino Biological	Flow cyt	18
CD3	Human	IS5033	Poly	Rabbit	Dako	IHC	44
Lysozyme	Human	A0099	Poly	Rabbit	Dako	IHC	44
CD20	Human	RB-9013-P	Poly	Rabbit	Thermo (Fisher)	IHC	44
CD79a	Human	HM57	IgG1, κ	Mouse	Dako	IHC	44
MHC-II	Human	TAL 1B5	IgG1, κ	Mouse	Dako	IHC	44
CD3	Human	PC3/188A	IgG1, κ	Mouse	Santa Cruz Biotech	Flow cyt	45
IFN-γ (capture Ab)	Cow	CC302	IgG1	Mouse	Bio-Rad	ELISpot/flow cyt	45
IFN-γ biotinylated (detection Ab)	Dog		Poly	Goat	R&D Systems		45, 46

a Abbreviations: HRP, horseradish peroxidase conjugate; TNF, tumor necrosis factor; Ab, antibody; Flow cyt, flow cytometry; IHC, immunohistochemistry.

To date, recombinant proteins representing a range of intrinsic, innate, and adaptive immune markers are under development, and some are already available from commercial sources (18, 19). These include type I and III interferons (IFNs), RIG-I and Toll-like receptors, cytokines, and chemokines, as well as cell surface markers for immune and nonimmune cells. In terms of adaptive immune responses, Kirchenbaum and Ross recently developed a monoclonal antibody against the ferret B cell receptor light chain that is useful in distinguishing kappa versus lambda B cell responses (20, 21). Enzyme-linked immunosorbent spot (ELISpot) and flow cytometric assays have been developed to quantify the isotypes of antibody-secreting cells (IgG or IgA) (22), pan-B cells (CD20^+^, CD79α^+^), and Ig^+^ B cells (18, 19). T cell phenotyping has been limited to quantification of overall CD3^+^ T cells, including CD4^+^ and CD8^+^ subsets, by flow cytometric assays and identification of antigen-specific effector responses by detecting IFN-γ secretion in flow-based intracellular cytokine secretion assays or ELISpot assays (18). An *in vivo* depletion of CD8 T cells using a cross-reactive human monoclonal antibody has been shown to delay influenza virus clearance (23). To increase our toolbox, the Centers for Excellence in Influenza Research and Surveillance (CEIRS) network has undertaken a large project to rapidly produce monoclonal antibodies and develop assays to support the universal influenza vaccine initiative (24). Antibodies in production include B cell markers (CD83, CD86, CD95, CD19, CD20, CD25, CD27, CD38, CD138, CXCR5, and FcR), T cell markers (CD4, CCR7, CD3e, CD40, CD40L, CD44, CD62L, CD69, CD103, PD-1, CXCR3, interleukin-7 receptor [IL-7R], and IL-15Ra) and others (CXCR4, CD140, IL-2, IL-21, and IL-4). These much-needed reagents will facilitate efforts to establish immunologic assays to interrogate the innate and adaptive immune responses to infection and vaccination at the level of detail that is routinely applied to studies of mouse or human immunology. Importantly, the ferret model will allow correlates of protection to be established after vaccination and infection in conjunction with transmission studies, which are not available in the mouse models. Additionally, the longer life span of the ferret relative to the mouse will allow analysis of the evolution of the immune response to sequential infection and/or vaccination (25), permitting more accurate modeling of the immune response in humans.

## WAYS FORWARD

While there has been exciting progress, much work remains to move the ferret model forward. Toward this goal, the CEIRS group has produced fibroblasts and primary nasal and tracheal epithelial cells and cell lines, established a repository of defined tissues and cell types (Table 3), and are working with the J. Craig Venter Institute to define the ferret major histocompatibility complex (MHC). An exciting achievement is the completion of the PacBio sequencing of the ferret MHC (Granger Sutton, personal communication). While these are important steps, the ultimate goal is to provide the biomedical research community with validated reagents and protocols they can trust to ensure the rigor and reproducibility in experiments utilizing the ferret model. In support of this goal, many of the reagents created through the CEIRS network will be made publicly available through the CEIRS Data Processing and Coordinating Center (DPCC) website (http://www.niaidceirs.org/resources/ceirs-reagents/).

**TABLE 3:** Current tissue repository

Tissue	Sample^a^	Sample forms	Sex	Comment
Lung	Brochioalveolar fluid		M	Influenza virus infected
	Upper right, middle right, lower right, upper left, lower left	Single-cell suspension; homogenate; whole tissue; Trizol; paraffin-embedded tissue	M and F	Influenza virus infected and noninfected
Blood	PBMC	Fluid; isolated cells; RNAlater	M and F	Influenza virus infected and noninfected
	Plasma		M	Noninfected
	Sera		M	Influenza virus infected and noninfected
Nasal fluid (wash)	NA	Fluid	M	Influenza virus infected and noninfected
Spleen	NA	Whole tissue; single-cell suspension; homogenate;	M and F	Influenza virus infected and noninfected
Trachea	NA	Whole tissue; single-cell suspension; homogenate; RNAlater	M and F	Influenza virus infected and noninfected
Mediastinal lymph node	NA	Whole tissue	M and F	Influenza virus infected

a PBMC, peripheral blood mononuclear cells; NA, not applicable.
